# Systematic Mutagenesis of Genes Encoding Predicted Autotransported Proteins of *Burkholderia pseudomallei* Identifies Factors Mediating Virulence in Mice, Net Intracellular Replication and a Novel Protein Conferring Serum Resistance

**DOI:** 10.1371/journal.pone.0121271

**Published:** 2015-04-01

**Authors:** Natalie R. Lazar Adler, Mark P. Stevens, Rachel E. Dean, Richard J. Saint, Depesh Pankhania, Joann L. Prior, Timothy P. Atkins, Bianca Kessler, Arnone Nithichanon, Ganjana Lertmemongkolchai, Edouard E. Galyov

**Affiliations:** 1 Department of Infection, Immunity and Inflammation, University of Leicester, Leicester, United Kingdom; 2 The Roslin Institute and Royal (Dick) School of Veterinary Studies, University of Edinburgh, Easter Bush, United Kingdom; 3 Biomedical Sciences, Defence Science and Technology Laboratory, Porton Down, United Kingdom; 4 School of Biosciences, Geoffrey Pope Building, University of Exeter, Exeter, United Kingdom; 5 The Centre for Research and Development of Medical Diagnostic Laboratories (CMDL), Faculty of Associated Medical Sciences, Khon Kaen University, Khon Kaen, Thailand; University of Toledo School of Medicine, UNITED STATES

## Abstract

*Burkholderia pseudomallei* is the causative agent of the severe tropical disease melioidosis, which commonly presents as sepsis. The *B*. *pseudomallei* K96243 genome encodes eleven predicted autotransporters, a diverse family of secreted and outer membrane proteins often associated with virulence. In a systematic study of these autotransporters, we constructed insertion mutants in each gene predicted to encode an autotransporter and assessed them for three pathogenesis-associated phenotypes: virulence in the BALB/c intra-peritoneal mouse melioidosis model, net intracellular replication in J774.2 murine macrophage-like cells and survival in 45% (v/v) normal human serum. From the complete repertoire of eleven autotransporter mutants, we identified eight mutants which exhibited an increase in median lethal dose of 1 to 2-log_10_ compared to the isogenic parent strain (*bcaA*, *boaA*, *boaB*, *bpaA*, *bpaC*, *bpaE*, *bpaF* and *bimA*). Four mutants, all demonstrating attenuation for virulence, exhibited reduced net intracellular replication in J774.2 macrophage-like cells (*bimA*, *boaB*, *bpaC* and *bpaE*). A single mutant (*bpaC*) was identified that exhibited significantly reduced serum survival compared to wild-type. The *bpaC* mutant, which demonstrated attenuation for virulence and net intracellular replication, was sensitive to complement-mediated killing via the classical and/or lectin pathway. Serum resistance was rescued by *in trans* complementation. Subsequently, we expressed recombinant proteins of the passenger domain of four predicted autotransporters representing each of the phenotypic groups identified: those attenuated for virulence (BcaA), those attenuated for virulence and net intracellular replication (BpaE), the BpaC mutant with defects in virulence, net intracellular replication and serum resistance and those displaying wild-type phenotypes (BatA). Only BcaA and BpaE elicited a strong IFN-γ response in a restimulation assay using whole blood from seropositive donors and were recognised by seropositive human sera from the endemic area. To conclude, several predicted autotransporters contribute to *B*. *pseudomallei* virulence and BpaC may do so by conferring resistance against complement-mediated killing.

## Introduction

*Burkholderia pseudomallei* is a motile Gram-negative bacillus which is a saprophyte of tropical and sub-tropical soils and water. It is the causative agent of melioidosis, a febrile illness with acute and chronic disease states, which often presents with sepsis. Melioidosis is associated with high mortality rates in endemic regions. For example, in North-Eastern Thailand it is the third most common cause of death from infectious disease and has a mortality rate of approximately 44% [1]. Cases of melioidosis have been reported outside of the traditional endemic regions of Thailand, Singapore, Malaysia and northern Australia, with other Asia-Pacific countries, the Caribbean, Central and South America as well as West and East Africa emerging on the map of worldwide disease distribution and prevalence [2]. Understanding of the molecular mechanisms of the pathogenesis of melioidosis has expanded considerably in recent years, primed in part by the availability of complete genome sequences. *B*. *pseudomallei* has a genome of >7 Mb comprising two chromosomes that encode many virulence factors, including capsule, lipopolysaccharide (LPS), flagella, quorum sensing systems and numerous protein secretion systems, including Type III and Type VI pathways [reviewed in [3,4]].

For bacterial pathogens that enter the blood, the magnitude of resistance of isolates to killing in serum associates with the severity of septicaemic disease [5]. The complement system, a component of the innate immune response, is a cascade of proteins which results in deposition of C3 on the microbial cell wall with the subsequent assembly of the C5b-C9 membrane attack complex that mediates cell lysis [6]. Complement can be activated via three independent pathways, the classical, lectin or alternative arms, which recognise microbes via immune complexes, polysaccharides, or foreign surfaces [7]. *B*. *pseudomallei* is resistant to the complement-mediated killing activity of normal human sera (NHS), being able to multiply in low levels of NHS (10–30% (v/v)) and exhibiting resistance to killing in up to 90% (v/v) NHS [8]. The complement cascade is activated by *B*. *pseudomallei* within five minutes, with deposition of C3 and the C5b-C9 membrane attack complex in a non-lytic manner [6]. Initial experiments demonstrated that *B*. *pseudomallei* predominately activated the alternative pathway of the complement system [6]. However, reduced C3 deposition was subsequently noted when the classical and lectin pathways were inactivated by EGTA/Mg^2+^ [9]. These data suggest that under varied conditions (i.e. Ca^2+^ levels), different arms of the complement pathways are activated by *B*. *pseudomallei*. Resistance to the alternative pathway has been attributed to the action of LPS O-antigen as seen in other Gram-negative organisms [8,10]. Yet while an LPS (*wbiI*) mutant is killed by the alternative arm of complement, lower levels of C3 deposition are observed compared to wild-type [9]. A double LPS and capsule (*wbiI* and *wcbB*) mutant was found to display a greater sensitivity to NHS than the LPS mutant alone (which itself displays an atypical capsule) and this phenotype could be abrogated by addition of purified capsular polysaccharide but not LPS [11]. But the acapsular mutant, which has normal LPS, demonstrated wild-type resistance to NHS [10,12] despite an increased deposition of C3 [9,11]. These data suggest that the capsule acts as a physical barrier for C3 deposition in a non-bactericidal manner, but when this barrier is absent other factors, i.e. LPS, are still able to provide resistance.

Autotransporters (ATs) comprise a large and diverse family of bacterial secreted and outer membrane proteins, which are present in many Gram-negative bacterial pathogens and play roles in numerous environmental and virulence-associated interactions including persistence, adhesion, serum resistance and biofilm formation. ATs utilise the Type V secretion mechanism, one of seven recognised secretion pathways in Gram-negative bacteria. A typical AT will have a 20–400 kDa passenger domain that contains the effector functions and a 10–30 kDa β barrel domain facilitating translocation across the outer membrane [13]. ATs can be further divided into classical ATs and trimeric autotransported adhesins (TAAs). TAAs are so named because each protein trimerises to form a single 12-stranded β barrel from their three 4-stranded β barrel domains. In many instances, the passenger domain of a typical AT functions as an adhesin [13,14]. The *B*. *pseudomallei* K96243 genome comprises genes encoding eleven predicted ATs, based on sequence similarity to known ATs [15,16], of which two are putative classical ATs and nine are predicted to be TAAs (reviewed in [16]; Table 1).

**Table 1:** Autotransporters of *B*. *pseudomallei* and their published functions.

**K96243 gene**	**Nomenclature**	**Function**	**Intracellular survival & replication**	**Virulence**	**Immunogenicity**
*Classical autotransporters*					
BPSL2237	BatA				
BPSS0962	BcaA		↓ invasion & plaques (A549) [17]		
*Trimeric autotransporter adhesions*					
BPSL1631	BpaC		↓ adhesion & invasion (A549) [15]; ↓ adhesion (NHBE) [18]	↓ liver survival (i.n. route) [15]; wt virulence (aerosol route) [18]	
BPSL1705	BoaB		↓ adhesion (A549) [15,19]		
BPSL2063	BpaB		↓ adhesion & invasion (A549) [15]		✓ [16]
BPSS0088	BpaD		↓ adhesion & invasion (A549) [15]		✓ [20]
BPSS0796	BoaA		↓ adhesion & invasion (A549) [15,19]		✓ [16]
BPSS0908	BpaE		↓ adhesion, invasion & plaques (A549) [15]		✓ [16,20]
BPSS1434	BpaA		↓ adhesion & invasion (A549) [15]		✓ [20]
BPSS1439	BpaF, BbfA ‡;^	Biofilm formation [21]	↓ invasion (A549) [15]		✓ [16]
BPSS1492	BimA^	Actin tails [22]	↓ actin tails (J774), plaques (A549) [23]		✓ [16,20]

‡ nomenclature used in this manuscript

^ name based on phenotypic characterisation

To date, the best studied of the *B*. *pseudomallei* ATs is BimA, a predicted TAA required for intracellular motility. BimA is located at the bacterial pole where it recruits and polymerises cellular actin to form actin tails which propel bacteria within, and between, host cells [22,23]. A second AT, BbfA, plays a role in biofilm formation; a *bbfA* mutant was found to be affected in its ability to form biofilms, as well as demonstrating attenuation for virulence via the intra-peritoneal route in the BALB/c murine melioidosis model [21]. However, a *B*. *pseudomallei* 1026b mutant in the gene encoding this same TAA, independently named *bpaF*, was found to display wild-type virulence via the intra-nasal route [15]. This result was part of a study of eight predicted TAAs (excluding BimA) which found that only BapC played a role in virulence via the intra-nasal route in the BALB/c mouse model. This study also demonstrated that mutants in *bpaA*, *bpaB*, *bpaC*, *bpaD*, *bpaE*, *bpaF*, *boaA* and *boaB* had reduced adhesion and/or invasion of A549 epithelial-like cell lines. Additionally, the *bpaE* mutant also showed reduced plaque formation in A549 cell monolayers [15]. Plaque formation in A549 cell monolayers is also impaired by mutation of *bimA*, though whether this reflects a lack of actin-based membrane protrusions or a defect in net intracellular replication is ill-defined [23]. The role of BoaA and BoaB in epithelial adhesion had been earlier demonstrated by the expression of recombinant BoaA and BoaB in a heterologous *E*. *coli* host resulting in an increased adhesive phenotype in the *E*. *coli* cells [19]. The role of BpaC in epithelial adhesion was confirmed by expression of recombinant BpaC in *E*. *coli*. The protein was observed to be localized on the bacterial surface by immunofluorescence microscopy [18]. A *bpaC* mutant of *B*. *pseudomallei* strain DD503 was found to exhibit reduced adhesion to normal human bronchial epithelium (NHBE) relative to its parent strain yet the *bpaC* mutant displayed no significant difference in virulence compared to the parent strain in the BALB/c mouse intra-tracheal aerosol model. BpaC-specific antibodies were detected in animals infected with the parent DD503 strain indicative of *in vivo* expression of BpaC [18]. Furthermore, screens using melioidosis patient sera found seven of the nine TAAs to be immunogenic, and can therefore be inferred to be expressed *in vivo* [16,20]. Only one study has focused on the classical ATs; a mutant lacking the putative protease BcaA was found to have a reduced invasion and plaque formation in A549 cell lines but no virulence phenotype [17].

As part of a systematic study on the ATs of *B*. *pseudomallei*, we have constructed insertion mutants in the genes encoding each of the eleven ATs of strain 10276. This includes the unnamed classical AT BPSL2237, which we have named BatA (*Burkholderia* autotransporter A). Our study extends upon the work of Campos *et al*., 2013 [15,17] and Lafontaine *et al*., 2014 [18] by investigating the complete AT repertoire for different *in vivo* and *in vitro* assays of virulence-associated phenotypes to elucidate the role of ATs in pathogenesis. Our data indicate that most of the putative ATs play a role in the virulence of *B*. *pseudomallei* 10276 in BALB/c mice when infection is delivered by the intra-peritoneal route (BcaA, BoaA, BoaB, BpaA, BpaC, BpaE, BbfA and BimA). Four mutants, all demonstrating attenuation for virulence, also exhibited reduced net intracellular replication in J774.2 macrophage-like cells (*bimA*, *boaB*, *bpaC* and *bpaE*). Additionally, we have identified that one predicted TAA, BpaC, plays a role in resistance to complement-mediated killing by 45% (v/v) NHS via the classical and/or lectin arm of the complement pathway.

## Materials and Methods

### Bacterial strains, plasmids, media and growth conditions

All bacterial strains and plasmids reported in these studies are listed in Table 2. The strains were routinely grown in Luria-Bertani (LB) broth with shaking or on LB with 1.5% (w/v) agar at 37°C. Cultures were supplemented with antibiotics and/or 10mM isopropyl-β-D-thiogalactopyranoside (IPTG) as required; kanamycin was used at a final concentration of 500 μg/ml, chloramphenicol at 100 μg/ml and tetracycline at 25 μg/ml.

**Table 2:** Strains and plasmids used in this study.

**Strain, plasmid or cell line**	**Characteristics**	**Insertion point / gene length** ^a^	**Reference or source**
*B*. *pseudomallei*:			
NCTC 10276	Clinical isolate (wild-type)		Dr T. Pitt (Public Health Laboratory Service, London, UK)
10276 (pME6032)	10276 harbouring the pME6032 plasmid		This study
10276 (pME6032-*bbfA*)	10276 harbouring the pME6032 plasmid containing a full-length copy of *bbfA*		[21]
10276 *bpaC*::pDM4 mutant	10276 harbouring a pDM4 single cross-over insertion in *bpaC*	638bp/ 3372bp	This study
10276 *bpaC*::pDM4 mutant (pME6032)	10276 harbouring a pDM4 single cross-over insertion in *bpaC* and the pME6032 plasmid		This study
10276 *bpaC*::pDM4 mutant (pME6032-*bpaC*)	10276 harbouring a pDM4 single cross-over insertion in *bpaC* and the pME6032 plasmid containing a full-length copy of *bpaC*		This study
10276 *bpaC*::pDM4 mutant (pME6032-*bbfA*)	10276 harbouring a pDM4 single cross-over insertion in *bpaC* and the pME6032 plasmid containing a full-length copy of *bbfA*		This study
10276 *bpaB*::pDM4 mutant	10276 harbouring a pDM4 single cross-over insertion in *bpaB*	715bp/ 3270bp	This study
10276 *batA*::pDM4 mutant	10276 harbouring a pDM4 single cross-over insertion in *batA*	426bp/1830bp	This study
10276 *bpaD*::pDM4 mutant	10276 harbouring a pDM4 single cross-over insertion in *bpaD*	1470bp/2023bp	This study
10276 *boaA*::pDM4 mutant	10276 harbouring a pDM4 single cross-over insertion in *boaA*	459bp/4959bp	This study
10276 *bpaE*::pDM4 mutant	10276 harbouring a pDM4 single cross-over insertion in *bpaE*	557bp/2313bp	This study
10276 *bcaA*::pDM4 mutant	10276 harbouring a pDM4 single cross-over insertion in *bcaA*	526bp/3393bp	This study
10276 *bimA*::pDM4 mutant	10276 harbouring a pDM4 single cross-over insertion in *bimA*	336bp/516bp	[22]
10276 *boaB*::pKNOCK-Kan mutant	10276 harbouring a pKNOCK-Kan single cross-over insertion in *boaB*	476bp/1606bp	This study
10276 *bpaA*::pKNOCK-Kan mutant	10276 harbouring a pKNOCK-Kan single cross-over insertion in *bpaA*	735bp/7902bp	This study
10276 *bbfA*::pKNOCK-Kan mutant	10276 harbouring a pKNOCK-Kan single cross-over insertion in *bbfA*	692bp/4590bp	[21]
*E*. *coli*:			
S17-1 λ*pir*	Conjugal donor for pDM4-based plasmids (λ*pir hsdR pro thi*; chromosomal integrated RP4-2 Tc::Mu Km::Tn*7*).		[24]
BL21 (DE3)	Protein expression strain (F^—^ *ompT gal dcm lon hsdS*_B_(r_B_^-^ m_B_^-^) λ (DE3 [*lacI lacU*V5-T7 gene 1 ind1 *sam7 nin5*])		[25]
Plasmids:			
pDM4	7.1kb, CmR, λ*pir*-dependent vector (*ori*R6K) with RP4 *oriT*		[26]
pDM4-*bpaC*	pDM4 containing a 509 bp *Xho*I- *Bgl*II internal fragment of *bpaC*		This study
pDM4-*bpaB*	pDM4 containing a 577 bp *Bam*HI- *Xba*I internal fragment of *bpaB*		This study
pDM4-*batA*	pDM4 containing a 357 bp *Bam*HI- *Xba*I internal fragment of *batA*		This study
pDM4-*bpaD*	pDM4 containing a 321 bp *Xba*I- *Eco*RI internal fragment of *bpaD*		This study
pDM4-*boaA*	pDM4 containing a 346 bp *Xho*I- *Bgl*II internal fragment of *boaA*		This study
pDM4-*bpaE*	pDM4 containing a 407 bp *Xho*I- *Bgl*II internal fragment of *bpaE*		This study
pDM4-*bcaA*	pDM4 containing a 364 bp *Xho*I- *Bgl*II internal fragment of *bcaA*		This study
pKNOCK-Kan	2.2 kb, Kan^R^, λ*pir*-dependent vector (*ori*R6K) with RP4 *oriT*		[27]
pKNOCK-Kan-*boaB*	pKNOCK-Kan containing a 407 bp *Bam*HI- *Xba*I internal fragment of *boaB*		This study
pKNOCK-Kan-*bpaA*	pKNOCK-Kan containing a 660 bp *Bam*HI- *Xba*I internal fragment of *bpaA*		This study
pME6032	9.8 kb, Tet^R^, pVS1 derived shuttle vector with IPTG inducible *p*tac promoter		[28]
pME6032-*bpaC*	pME6032 containing a full-length copy of *bpaC* (3755bp)		This study
pGEX4T1	5.0 kb, Amp^R^, N-terminal GST fusion protein vector with IPTG inducible *p*tac promoter and thrombin protease cleavage site		GE Healthcare
pGEX4T1-*bpaE*	pGEX4T1 containing a 1575 bp passenger domain fragment of *bpaE*		This study
pGEX4T1-*bpaC*	pGEX4T1 containing a 2241 bp passenger domain fragment of *bpaC*		This study
pGEX4T1-*bcaA*	pGEX4T1 containing a 2145 bp passenger domain fragment of *bcaA*		This study
pGEX4T1-*batA*	pGEX4T1 containing a 933 bp passenger domain fragment of *batA*		This study

^a^ Gene length (bp) includes the region encoding the N-terminal passenger domain which would be cleaved upon protein transport across the inner membrane.

### Recombinant DNA techniques

Plasmid DNA isolation, PCR fragment purification and gel extraction were performed using Bioline kits (London, UK) as per manufacturer’s instructions. Genomic DNA was isolated from *B*. *pseudomallei* as previously described [29]. Restriction endonucleases and DNA modifying enzymes (New England Biolabs, Hitchin, UK) were used as per manufacturer’s instructions. PCR amplifications were performed using Advantage Taq (Clontech, Saint-Germain-en-Laye, France) or Taq DNA polymerase (New England Biolabs, Hitchin, UK) in accordance with manufacturer’s instructions. DNA sequencing was carried out at the Protein and Nucleic Acid Laboratory, University of Leicester.

The single cross-over insertion mutants were generated using either pDM4 [26] or pKNOCK-Kan [27] as described previously and in Table 2. Briefly, an internal fragment of the gene encoding the AT was amplified by PCR from *B*. *pseudomallei* 10276 using primers with engineered restriction enzyme sites (S1 Table). The PCR products containing the internal fragment were digested with the relevant enzymes, alongside the vector, and these were ligated together. The recombinant plasmids were introduced into *B*. *pseudomallei* 10276 via conjugation from *E*. *coli* S17-1 λ*pir* [27] and inactivation of the gene encoding the AT was confirmed by PCR and sequencing of the insertion boundaries.

To allow for *in trans* complementation of the *bpaC* mutant, the full-length *bpaC* gene was amplified by PCR from *B*. *pseudomallei* 10276 using primers with engineered restriction enzyme sites (S1 Table). The PCR product containing the *bpaC* gene was digested with *Eco*RI and *Xho*I and ligated into similarly digested pME6032 [28] under the control of a *lac* promoter to form pME6032-*bpaC* (pME-*bpaC*).

For protein expression, a gene fragment encoding the passenger domain (without N-terminal signal peptide or C-terminal membrane domain) was amplified by PCR from *B*. *pseudomallei* 10276 using primers with engineered restriction enzyme sites (S1 Table). The protein expression vector, pGEX4T1 (GE Healthcare, Little Chalfont, UK), was selected to construct N-terminal recombinant glutathione-S-transferase (GST)-fusion proteins under the induction of IPTG. The PCR products containing the passenger domain were digested with the relevant enzymes, alongside the vector, and these were ligated together (Table 2). The recombinant plasmids were transformed into *E*. *coli* BL21 (DE3) [25]. Recombinant fusion protein expression vectors were sequenced to confirm PCR accuracy.

All the genetic modifications detailed in this manuscript were performed with the consent of the UK Health and Safety Executive and University of Leicester and Institute for Animal Health Biological & GM Safety Committees. The work was done in full compliance with all relevant risk assessments and the requirements of the Advisory Committee of Dangerous Pathogens for work with hazard group 3 micro-organisms and Home Office requirements for the storage and use of Schedule 5 biothreat agents.

### Ethics Statement

All investigations involving animals were carried out under a UK Home Office Project Licence, according to the requirements of the Animal (Scientific Procedures) Act 1986. Ethical approval was granted by our local (Defence Science and Technology Laboratory, University of Leicester) ethical review process according to the requirements of the Animal (Scientific Procedures) Act 1986. Animals were monitored a minimum of twice daily, or more frequently once disease occurred. Clinical signs characteristic of disease, such as ruffled coats, hunched posture, lethargy, eye problems, neurological complications and reduced mobility were used to score disease severity as mild, moderate or severe. Animals that reached severe disease (Defence Science and Technology Laboratory) or a moderate lethargic state (University of Leicester) were culled via cervical dislocation according to schedule 1 of the Animal (Scientific Procedures) Act 1986. Animals that showed neurological complications or were immobile were also immediately culled, regardless of other observed clinical signs.

### Assessment of virulence in mice

For each of the eleven AT mutants and the wild-type 10276 parent strain, an initial evaluation of virulence was performed by challenging groups of six BALB/c mice (6–8 weeks old) via the intra-peritoneal route with 10^5^ CFU. Mice were monitored for signs of disease over a 35 day period and humanely culled when pre-defined end-points were reached by cervical dislocation according to Schedule 1 of the Animal (Scientific Procedures) Act 1986.

The eight AT mutants which displayed attenuation for virulence relative to the wild-type 10276 strain were subsequently tested in six groups of five female BALB/c mice (6–8 weeks old). Mice were separately challenged by the intra-peritoneal route at doses rising in ten-fold increments from 10^2^ to 10^7^ CFU (five mice per dose per strain). Mice were monitored for signs of disease over a 35 day period and humanely culled when pre-defined end-points were reached by cervical dislocation according to Schedule 1 of the Animal (Scientific Procedures) Act 1986. The median lethal dose was determined using the Reed and Muench calculation based on cumulative death.

### Analysis of net intracellular replication in J774.2 murine macrophage-like cells

The intracellular survival and/or replication of *B*. *pseudomallei* strains in monolayers of the macrophage-like J774.2 cell line was assessed as previously reported [21]. Briefly, J774.2 cell monolayers were infected with *B*. *pseudomallei* at an MOI of 10 for 1 h then washed in phosphate-buffered saline (PBS) and incubated for a further 15 h in Dulbecco’s modified Eagle medium (DMEM) + 10% (v/v) heat inactivated foetal calf serum (FCS) containing gentamicin and spectinomycin (192 μg/ml and 384 μg/ml) to kill extracellular bacteria. Growth media was supplemented with 25 μg/ml tetracycline and 10 mM IPTG as required. J774.2 cells were lysed with 0.2% (v/v) Triton X-100 and viable bacteria were enumerated by incubation on LA agar plates. Data for the eleven AT mutants (the output CFU of the mutant strain divided by the input CFU) was normalised against the wild-type data and the normalised values from duplicate samples in five independent experiments were expressed as the mean and standard error of the mean; statistical significance was analysed using Student’s *t* test.

### Serum resistance assays

NHS was collected from healthy adult volunteers (n = 2) with informed written consent in accordance with the requirements of the Human Tissue Act (2004) with the approval of the University of Leicester Local Ethical Review Committee which reviewed both the collection and consent procedures. Serum resistance assays were performed as previously reported [6]. Briefly, *B*. *pseudomallei* strains were added at 10^6^ CFU to 45% (v/v) NHS in PBS and bacterial survival was enumerated after 3 h by plating onto LA. Serum was supplemented with 25 μg/ml tetracycline and 10 mM IPTG during plasmid-mediated *trans*-complementation. Assays were performed using duplicate samples over four independent experiments or quadruplicate samples over three independent experiments (depending on the number of samples per experiment).

### Polysaccharide staining

The *bpaC* mutant and its parent wild-type 10276 strain were assessed for the presence of polysaccharide capsule using immunofluorescence microscopy [30] and LPS using silver staining [31] and immunoblotting [32]. For capsule, mid-logarithmic phase cultures of the *B*. *pseudomallei* strains were air-dried onto microscope slides and fixed overnight with 4% (w/v) PFA prior to staining for 1 h with the type I O-PS capsule monoclonal antibody 4V1H12 (1 μg/ml) followed by goat anti-mouse IgG Alexa Fluor 488 (Molecular Probes, Invitrogen, Paisley, UK) at a 1:250 dilution for 1 h. Stained bacteria were observed using immunofluorescence microscopy and a minimum of nine fields of view were observed. For LPS, overnight cultures of the *B*. *pseudomallei* strains were pelleted by centrifugation and the bacterial cells lysed by boiling for 10 minutes in 300 μl 1x SDS-PAGE sample buffer prior to Proteinase K (0.5 mg/ml) treatment for 1 h at 60°C. Samples were loaded in duplicate onto 17% (v/v) polyacrylamide Tris-buffered gels without SDS. Carbohydrates were visualised by silver staining of the oxidised gel (0.35% (v/v) periodic acid treatment) using SilverQuest (Invitrogen, Paisley, UK) as per manufacturer’s instructions. Additionally, the gel was transferred onto Hybond ECL nitrocellulose membranes (GE Healthcare, Little Chalfont, UK) by semi-dry blotting (Sigma-Aldrich) as per manufacturer’s instructions. The membrane was blocked overnight in 0.5% (w/v) milk powder followed by primary antibody detection with anti-*B*. *mallei* LPS monoclonal antibody (AbCam, Cambridge, UK) and secondary antibody detection with anti-mouse IgG AP-linked antibody (Cell signalling Technology, Hitchin, UK). Antibodies were used at a dilution of 1:2000 for 1 h.

### RNA Isolation and analysis

RNA was isolated from *B*. *pseudomallei* strains using the TRIzol reagent (Invitrogen, Paisley, Scotland) as per manufacturers’ instructions. Briefly, cells were harvested from mid-log cultures at an OD_650_ of 1.0 and resuspended in 1 ml TRIzol. Proteinaceous impurities were removed by duplicate chloroform extraction steps and RNA was precipitated with 0.6 volume isopropanol, washed with 70% (v/v) Ethanol and resuspended in 100 μl H_2_O. To remove contaminating DNA, RNA (1 μg) was incubated with 6U DNase in 10x buffer (Fisher, Loughborough, UK) in a total volume of 100 μl for 60 minutes at 37°C. RNA was then purified using RNeasy columns (Qiagen, Hilden, Germany) as per the manufacturer’s instructions. The DNase treatment was repeated a second time prior to reverse transcription.

Reverse transcription of RNA (500 ng) to obtain cDNA was performed using 50 ng random hexamers and RevertAid reverse transcriptase (Fermentas, Loughborough, UK) as per manufacturers’ instructions. Negative control reactions were performed concurrently with the reverse transcriptase omitted to confirm complete removal of gDNA from each sample. cDNA was then used in PCR amplifications as described for recombinant DNA techniques above. Resulting PCR products were visualised on 0.8% (w/v) agarose gel using a Syngene G:Box with its proprietary GeneSnap v7.12.02 software (Syngene, Cambridge, UK). Duplicate biological samples were tested and densitometry was performed against the gDNA (15 ng) positive control.

### Protein expression and purification

Recombinant N-terminal GST fusion proteins were expressed in *E*. *coli* BL21 (DE3) cells. Bacterial strains harbouring pGEX4T1 constructs were grown in 200 ml LB and protein expression induced with 1 mM IPTG at an OD_650_ of 0.6 to 0.8 at 37°C for 6 h. Cells were harvested by centrifugation and lysed in lysozyme (10 mg/ml) prior to sonication. Insoluble inclusion bodies were purified by sequential wash and centrifugation steps in 2 M urea followed by overnight incubation at -20°C. The resulting supernatant was subjected to dialysis against PBS to remove urea. Soluble recombinant fusion proteins were quantified by the Coomassie (Bradford) Protein Assay kit (Thermo Scientific, Loughborough, UK) using a BSA standard curve as per manufacturer’s instructions and proteins were adjusted to a final concentration of 1 mg/ml.

### Immune recognition of recombinant proteins

The recombinant N-terminal GST fusion proteins were used as antigens to test the immune response against ATs. The four antigens (0.3 μg/ml) were used to restimulate lymphocytes in whole blood from seropositive donors (WB) (n = 2), alongside phytohaemagglutinin (PHA) and paraformaldehyde (PFA)-fixed *B*. *pseudomallei* strain K96243 positive controls. Whole blood was collected from healthy adult volunteers in the (endemic) Khon Kaen area of Northeastern Thailand with informed written consent; the study, including the consent form, was reviewed and approved by the Khon Kaen University Ethics Committee for Human Research. Seropositive donors were then identified by using the indirect haemagglutination assay; a titer of ≥ 40 was considered seropositive. The IFN-γ response of WB was measured by ELISA as previously described [33]. IFN-γ production indicates lymphocyte activation (memory) in response to the specific antigens tested.

The four antigens were also tested for recognition by seropositive sera from melioidosis endemic area using an indirect sandwich ELISA. A 96-well plate was coated with 3 μg/ml crude *B*. *pseudomallei* antigen extracted from clinical isolates as described previously [34] or 10 μg/ml of each recombinant protein; uncoated wells served as a background absorbance control. The plate was probed with a 1:300 dilution of plasma from healthy seropositive volunteers (n = 15) prior to detection with a 1:10,000 dilution of biotinylated anti-human IgG and Streptavidin-horse radish peroxidase. The ELISA plate was read at OD_450_; results were represented by absorbance index (test absorbance minus the background divided by the background). Duplicate experiments were performed; statistical significance was calculated using one way ANOVA with Tukey’s post test.

## Results and Discussion

### ATs play a role in the virulence of *B*. *pseudomallei*

As part of a systematic study on the ATs of *B*. *pseudomallei*, we constructed and validated insertion mutants in all eleven predicted ATs of the virulent strain 10276 as detailed in Materials and Methods. Predictions were based on published searches (reviewed in [35]). All eleven mutants were tested alongside their isogenic parent 10276 strain and found to display wild-type levels of *in vitro* growth in LB media (data not shown). To assess the role of ATs in the virulence of *B*. *pseudomallei*, these mutants and the wild-type 10276 parent strain were tested for virulence in a BALB/c mouse model of melioidosis. An initial single dose virulence screen demonstrated attenuation for virulence for eight AT mutant compared to the wild-type strain (data not shown). Therefore, six groups of five mice were separately challenged by the intra-peritoneal route with rising doses of each of these eight AT mutants. The median lethal dose (MLD) of the eight AT mutants or wild-type 10276 was determined by the using the Reed and Muench calculation based on cumulative lethal dose. Compared to the parent 10276 strain (MLD 1.6 x 10^4^ CFU), eight of the AT mutant strains exhibited an increase in median lethal dose of between 1 to 2-log_10_; *bpaA* (1.52 x 10^6^ CFU), *bpaC* (1.02 x 10^6^ CFU), *bpaE* (6.34 x 10^5^ CFU), *boaB* (6.13 x 10^5^ CFU), *bcaA* (5.78 x 10^5^ CFU), *bbfA* (2.09 x 10^5^ CFU), *boaA* (1.6 x 10^5^ CFU) and *bimA* (1.6 x 10^5^ CFU) (Table 3). These data reveal that the majority of ATs, with the exception of BatA, BpaB and BpaD, play a role in the virulence of *B*. *pseudomallei*.

**Table 3:** Virulence of *B*. *pseudomallei* autotransporter mutants in a murine model of melioidosis.

**K96243 gene**	**Nomenclature**	**Function**	**Attenuation (MLD compared to wild-type)**
*Wild-type 10276*			
-	-	-	1.6 x 10^4^ CFU
*Classical autotransporters*			
BPSL2237	BatA		N/D
BPSS0962	BcaA		✓ (5.78 x 10^5^ CFU; 36-fold)
*Trimeric autotransporter adhesins*			
BPSL1631	BpaC		✓ (1.02 x 10^6^ CFU; 64-fold)
BPSL1705	BoaB		✓ (6.13 x 10^5^ CFU; 38-fold)
BPSL2063	BpaB		N/D
BPSS0088	BpaD		N/D
BPSS0796	BoaA		✓ (1.6 x 10^5^ CFU; 10-fold)
BPSS0908	BpaE		✓ (6.34 x 10^5^ CFU; 40-fold)
BPSS1434	BpaA		✓ (1.52 x 10^6^ CFU; 95-fold)
BPSS1439	BbfA	Biofilm formation [21]	✓ (2.09 x 10^5^ CFU; 13-fold)
BPSS1492	BimA	Actin polymerisation [22]	✓ (1.6 x 10^5^ CFU; 10-fold)

N/D = not done

Our virulence data diverges from previously published data from the two studies by Campos *et al*., 2013 [15,17], where attenuation was only detected for the *bpaC* mutant within the liver of the intra-nasal mouse model. However, consistent with these observations, the *bpaC* mutant demonstrated the second highest median lethal dose in our experiments. The studies of Campos *et al*., 2013 [15,17] involved infection via the intra-nasal route, which resulted in an early acute disease with bacterial burdens of 10^5^ to 10^8^ CFUs in the liver and spleen by 48 h. As acknowledged by the authors, this highly acute model is unlikely to detect moderate attenuation levels observed in our animal model over a longer time course, where intra-peritoneal dosing produces morbidity several weeks after challenge rather than several days as with intra-nasal administration (reviewed in [36]).

The intra-tracheal aerosol infection data for the *bpaC* mutant obtained by Lafontaine *et al*., 2014 [18], also represents an early acute disease although this route of infection has been shown to target the lungs [37] rather than the nasal cavity [38] tropism noted for intra-nasal infection. The difference in target site for primary infection may explain the why the *bpaC* mutant displayed a median lethal dose similar to its parent strain via the intra-tracheal aerosol route [18]. It is important to note that the independent *bpaC* mutants were constructed using different methods (deletion [18] versus insertion [15] mutants). Insertion mutants can result in the expression of truncated proteins, which can potentially cause attenuation. To minimize any possible negative dominant effects, our insertion mutants were designed so that should protein expression occur, only a significantly truncated protein (representing approximately 24% of the full-length AT) could be translated (Table 2). It is also important to remember, that any potential truncated ATs would lack their C-terminal translocator domain; thus, would be retained in the periplasmic space.

An additional difference between these previous studies is the choice of strain background; while both groups used strain 1026b, each study used a 1026b mutant lacking different antibiotic resistance genes. In the study by Campos *et al*., 2013, Bp340 [39], a 1026b background strain with a deletion in the genes encoding the antibiotic efflux pump *bpeAB*-*oprB* was used while in the study of Lafontaine *et al*., 2014, DD503 [40], a 1026b background strain with a deletion in the genes encoding a second antibiotic efflux pump *amrR-oprA* was used. The DD503 strain has been previously reported to display an increased median lethal dose of 2.5 log_10_ relative to its 1026b wild-type parent in the whole body aerosol model in BALB/c mice [41]. Conversely, the median lethal dose for 1026b via the intra-tracheal aerosol route previously published by Lafontaine *et al*., 2014 [37] was 0.5 log_10_ higher than the median lethal doses of DD503 and the *bpaC* mutant. Contradictory pleiotropic phenotypes for *bpeAB*-*oprB* mutants in the 1026b and KHW strains have also been reported including defects in biofilm formation, quorum sensing and infection of epithelial and macrophage cell lines [39,42]. Therefore, in addition to the suggestion of Lafontaine *et al*., 2014 [18] that testing of various infection models is required for a full understanding of the role of BpaC in pathogenesis, further investigation of the strain-dependent effects should be assessed.

Five of the genes encoding ATs occur within operons as defined by the comprehensive transcriptome study of Ooi *et al*., 2013 [43] (*bcaA*, *bpaB*, *bpaE*, *bbfA* and *bimA*). Of these, four exhibit attenuation of virulence. The *bbfA* mutant has been previously identified to play a role in biofilm formation and virulence and both of these mutant phenotypes were rescued by *trans*-complementation implying a lack of polarity [21]. Additionally, the defect in actin-based motility of a *bimA* mutant can be fully rescued by plasmid-mediated *trans*-complementation within J774.2 macrophage-like cells and the original annotation of the locus shows *bimA* to be the last predicted gene in an operon [22]. The *bcaA* and *bpaE* genes are the first in predicted two-gene operons and we cannot preclude the possibility that the downstream genes (respectively predicted to encode a hypothetical protein and an OmpA family protein) may contribute to the observed phenotypes of the insertion mutants in mice.

### Four ATs have a significant effect on net intracellular replication of *B*. *pseudomallei* in J774.2 macrophage-like cells

**Fig 1:**
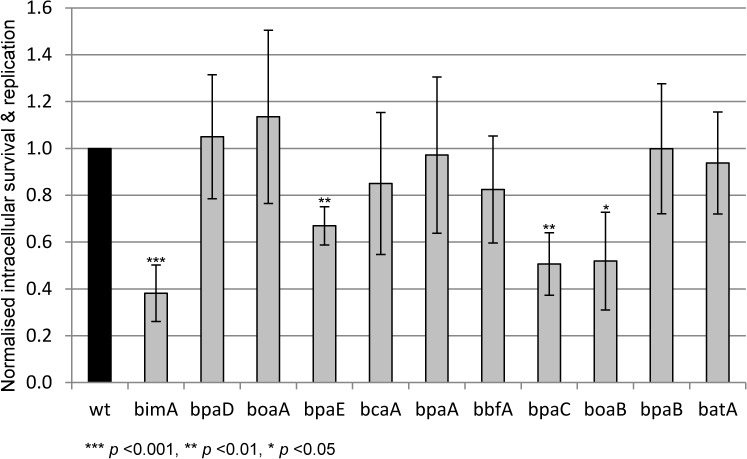
Net intracellular replication of the *B*. *pseudomallei* AT mutants in J774.2 macrophage-like cells. Viable intracellular bacteria were enumerated (output divided by input CFU) for the duplicate experimental samples of each of the AT mutants and the data was normalised against the wild-type parent strain. Data from five experiments was expressed as the mean and standard error of the mean; statistical significance was analysed using Student’s *t* test.

Previous studies have demonstrated that most of the *B*. *pseudomallei* ATs play a role in adhesion, invasion and/or plaque formation in epithelial cell lines [15,17–19]. However, only *bimA*, *bbfA* and *bpaC* mutants of *B*. *pseudomallei* have been tested for intracellular survival in phagocytic cells [21,23] with only the *bimA* mutant found to display reduced survival and/or replication. Therefore, we assessed the role of ATs in the survival and/or replication of *B*. *pseudomallei* within the macrophage-like J774.2 cell line as detailed in Materials and Methods. Four AT mutant strains demonstrated significantly reduced net intracellular replication relative to the wild-type: *bimA* (0.38± 0.12, *p* = 0.0009), *bpaE* (0.67± 0.08, *p* = 0.0037), *bpaC* (0.51± 0.13, *p* = 0.0061) and *boaB* (0.52± 0.21, *p* = 0.0500) (Fig. 1). Data were consistent with our earlier data for the *bbfA* mutant [21] and unpublished observations for the *bimA* mutant recorded by Stevens *et al*., 2005 [22]. However, our results were not in accordance with the published data for a *bpaC* mutant tested by Campos *et al*., which did not demonstrate a defect in net intracellular survival of J774A.1 macrophage-like cells in their study [18]. Differences in the macrophage cell line, assay time (8 h [18] versus our 16 h) or bacterial strain variation may explain this disparity.

The four AT mutant strains which demonstrated reduced net intracellular survival were not those which displayed the highest levels of attenuation of virulence in mice (Table 3). The importance of the intracellular phase of infection has not been elucidated and such results highlight the need for in-depth studies into this nascent area. The four mutants attenuated for net intracellular replication, *bimA*, *bpaE*, *bpaC* and *boaB*, are all TAAs, although these are overrepresented in the *B*. *pseudomallei* genome [35]. Though *bimA* has been demonstrated to play a role in plaque formation [22], we present the first compelling evidence of an important role for this factor in *B*. *pseudomallei* virulence and net intracellular growth, in contrast with the absence of attenuation of a *B*. *mallei bimA* mutant in a Syrian hamster model of acute glanders [44]. Mutants lacking the three other TAAs required for net intracellular replication (*bpaE*, *bpaC* and *boaB*) were examined by fluorescence microscopy and found to form normal actin tails during infection of macrophage-like cells (data not shown).

### A *B*. *pseudomallei bpaC* mutant is sensitive to complement-mediated killing by normal human serum via the classical and/or lectin pathway of the complement system

**Fig 2:**
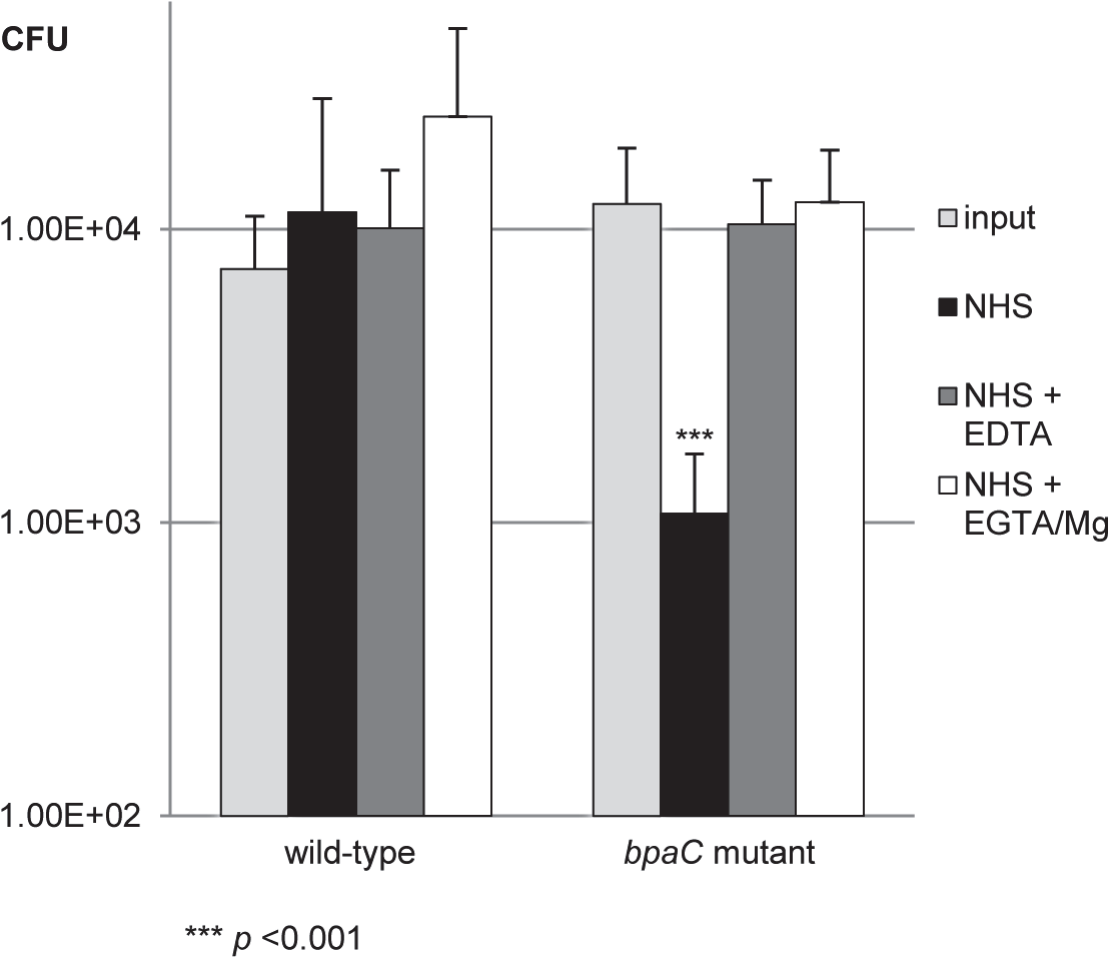
The *bpaC* mutant is sensitive to complement-mediated killing. The *bpaC* mutant demonstrates a 1.1-log_10_ reduced survival in 45% (v/v) NHS after 3 h incubation relative to the parent strain (1.08 x 10^3^ CFU versus the 1.22 x 10^4^ CFU input, *p* = 0.0003). This sensitivity could be abrogated by the addition of 10 mM EDTA or 10 mM EGTA, 5 mM MgCl_2_. Graphs show mean results with error bars displaying standard error of the mean; statistical significance was calculated using Student’s *t* test.

The reported functions of ATs include a role in serum resistance in a variety of organisms, including the related respiratory pathogens *Burkholderia cenocepacia* [45] and *Bordetella pertussis* [46]. To investigate the role of ATs in serum resistance, the eleven *B*. *pseudomallei* AT mutants and the wild-type 10276 parent strain were tested for survival in 45% (v/v) NHS after 3 h incubation (data not shown and Fig. 2). Only one *B*. *pseudomallei* strain, the *bpaC* mutant, demonstrated significantly reduced survival in serum. These data are not in accordance with the previously published *bpaC* mutant [18] which did not demonstrate a defect in bactericidal activity of 50% (v/v) NHS. However, in addition to using a different bacterial strain, the authors of this report enumerated bacteria at 30 minutes post-exposure [18] rather than 180 minutes post-exposure as in the present study. Congruently, we found that the serum sensitivity phenotype of the *bpaC* mutant was not statistically significant at 30 minutes (data not shown).

To further evaluate the ability of *B*. *pseudomallei* to resist the complement-mediated killing of NHS, the wild-type and *bpaC* mutant strains were assessed for survival in NHS with and without the addition of 10 mM EDTA (inactivation of complement activity) or 10 mM EGTA, 5 mM MgCl_2_ (inactivation of the classical and lectin arms of the complement pathway). The *bpaC* mutant exhibited a 1.1-log_10_ reduction in survival compared to the wild-type in 45% (v/v) NHS after 3 h incubation relative to the parent strain (1.08 x 10^3^ CFU versus the 1.22 x 10^4^ CFU input, *p* = 0.0003) (Fig. 2). This serum sensitivity was abolished by disruption of all complement as well as by specific inactivation of the classical and/or lectin pathway (Fig. 2A).

Both polysaccharide capsule and the LPS are important for serum resistance; therefore, it is important to ensure that disruption of the *bpaC* gene did not result in any disruption to these polysaccharides. The *bpaC* mutant and its parent wild-type 10276 strain were assessed for the presence of capsular material using immunofluorescence microscopy and for LPS using silver staining and immunoblotting as detailed in Materials and Methods. Thus, we can confirm that both polysaccharide capsule and LPS, important serum resistance factors, are expressed in the *bpaC* mutant (S1 Fig.).

The mechanism of action of ATs mediating serum resistance varies greatly. The prototypical TAA, YadA from *Yersinia* spp., binds serum complement regulatory factors from both the alternative and classical/ lectin pathway; factor H and C4 binding protein respectively [47,48]. The serum resistance TAAs of *Moraxella catarhalis*, UspA1 and UspA2, also bind C4 binding protein [49], in addition to factor C3d [50]. *Bordetella pertussis* has multiple ATs involved in serum resistance, including BrkA, BapC and Vag8. BrkA and Vag8 both inhibit the classical and/or lectin pathway. BrkA prevents C4 deposition via an uncharacterised mechanism [51] while Vag8 binds to the C1 inhibitor in a BrkA-independent manner [46]. BapC has an additive effect on BrkA serum resistance but its mechanism of action is unknown [52]. The *Haemophilus ducreyi* TAA DsrA mediates serum resistance by binding IgM [53]. *Escherichia coli* also encodes a family of five TAAs (EibA, C, D, E and F) which bind the Fc fragment of IgG and/or IgA [54]. In *B*. *pseudomallei*, the observed serum resistance phenotype of the *bpaC* mutant could be due to BpaC-mediated interaction(s) with classical and/or lectin pathway regulators, for example C1 inhibitor or C4 binding protein, or by the binding of non-specific immunoglobulins [7]. Further studies are required in order to elucidate the target complement factor, as well as the mechanism and required domains of this BpaC-mediated serum resistance.

### Complementation of the serum sensitivity and intracellular survival phenotypes of the *bpaC* mutant

**Fig 3:**
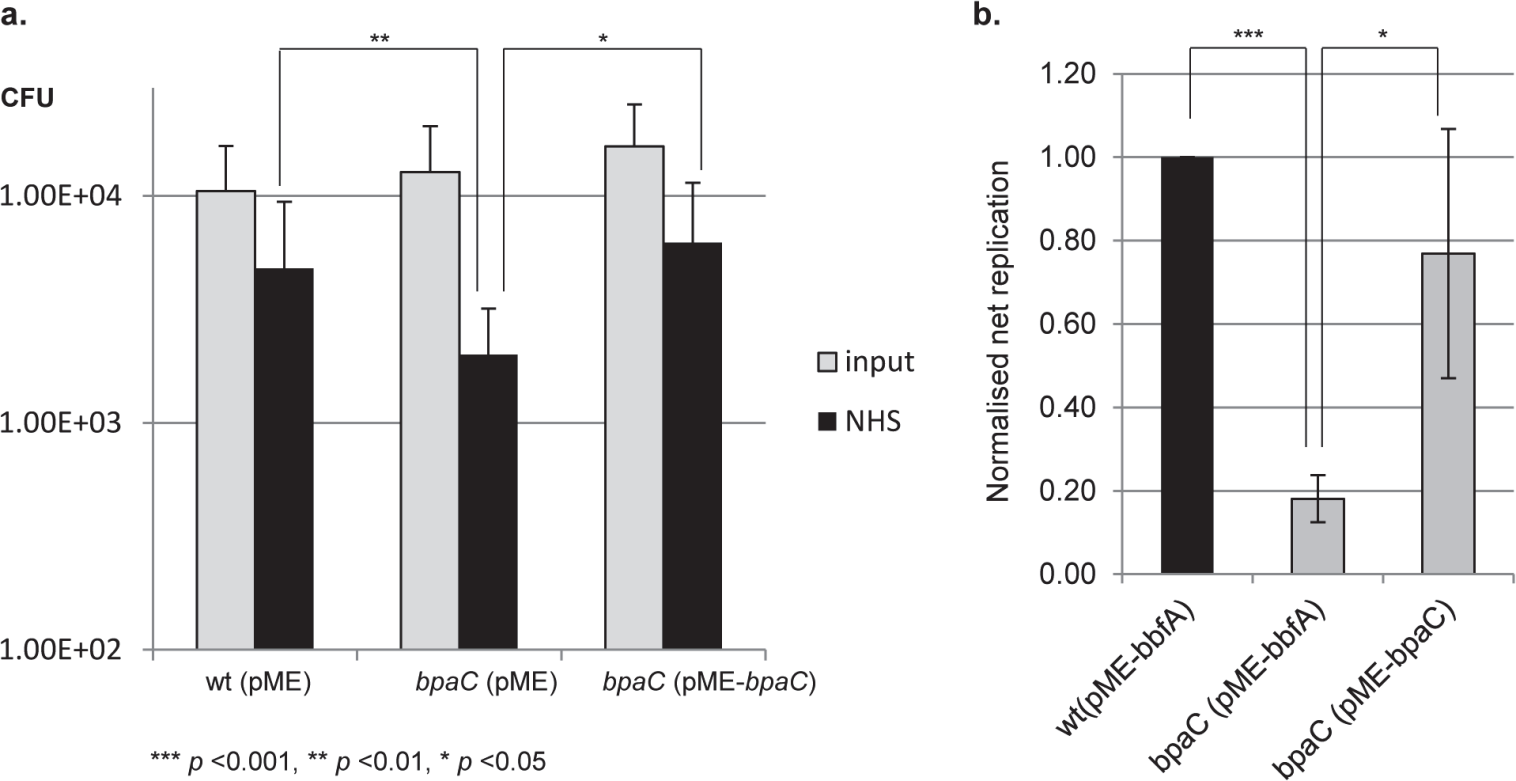
Complementation of the *bpaC* mutant serum sensitivity and intracellular survival phenotypes. a. Complementation of the *bpaC* mutant with a full-length copy of the *bpaC* gene expressed *in trans* by the inducible pME6032 vector (pME-*bpaC*) abrogated the serum sensitive phenotype seen for the *bpaC* mutant harbouring empty pME6032 vector (pME). Graphs show mean results with error bars displaying standard error of the mean; statistical significance was calculated using Student’s *t* test. b. Complementation of the *bpaC* mutant with a full-length copy of the *bpaC* gene expressed *in trans* by the inducible pME6032 vector (pME-*bpaC*) partially complemented the defect in net intracellular replication seen for the *bpaC* mutant harbouring pME6032 vector with a full-length copy of the *bbfA* gene (pME-*bbfA*). Graphs show normalised mean results with error bars displaying standard error of the mean; statistical significance was calculated using Student’s *t* test.

To confirm that the observed human serum sensitivity was due to the disruption of *bpaC*, the *bpaC* mutant was complemented *in trans* with the inducible pME6032 vector containing a full-length copy of the *bpaC* gene. Additionally, *B*. *pseudomallei* wild-type and a *bpaC* strain harbouring empty pME6032 (pME) vector were used as controls to account for any potential effects of the plasmid *per se* on serum resistance. Serum assays were repeated as described previously and a significant reduction in serum resistance of the *bpaC* (pME) strain was observed relative to the wild-type (pME) strain. However, serum resistance by the complemented *bpaC* (pME-*bpaC*) strain was restored to wild-type levels in the presence of inducer (Fig. 3A). Therefore, it can be concluded that BpaC is a novel serum resistance factor that mediates resistance to the classical and/or lectin arms of the complement pathway.

Following the successful complementation of the *bpaC* mutant for *in vitro* serum resistance assays, we repeated the macrophage assays as described previously. The *B*. *pseudomallei* wild-type and *bpaC* strains harbouring empty pME6032 (pME) vector resulted in a loss of fitness for both bacterial strains under the intracellular experimental conditions. Therefore, these controls were replaced with a *B*. *pseudomallei* wild-type and a *bpaC* strain harbouring the previously reported pME6032 plasmid containing a full-length copy of the *bbfA* gene (pME-*bbfA*) [21]. A significant reduction in net intracellular survival of the *bpaC* (pME-*bbfA*) mutant strain was observed (0.18± 0.06, *p*<0.001 normalised against the wild-type strain harbouring pME-*bbfA*) (Fig. 3B), supporting the effect of *bpaC* mutation seen in Fig. 1. The net intracellular replication of the complemented *bpaC* (pME-*bpaC*) was largely restored in the presence of inducer (0.71± 0.33, *p* = 0.411 relative to the wild-type) (Fig. 3B). Therefore, it can be concluded that BpaC has a role in net intracellular survival of *B*. *pseudomallei* 10276 in phagocytic cells.

Finally, an attempt at *in trans* complementation of the *bpaC* mutant virulence phenotype was performed using the competitive *in vivo* growth assay method successfully used to confirm a direct role for BbfA in virulence [21]. Unfortunately, the pME6032 plasmid harbouring a full-length copy of *bpaC* complementation vector did not rescue the *bpaC* mutant phenotype despite negligible loss of plasmid over the 24 h experimental period (data not shown). As *bpaC* is not predicted to be encoded in an operon, polar effects of the insertion are unlikely. However, it is also possible that the cloned 117 bp upstream region does not contain the full promoter region(s) for expression. Given this assumption, *bpaC* gene expression would need to be driven by exogenous IPTG. Induction with IPTG occurred via *in vitro* addition during the serum assays and macrophage uptake from supplemented cell culture media for the macrophage assays but was not present during *in vivo* animal studies. Experimental results for the *in vitro* serum assay and intracellular macrophage assay concur with this hypothesis as complementation levels were significantly higher in the serum assays for which IPTG was more readily accessible (Fig. 3). To test this hypothesis, we incubated the *bpaC* mutant harbouring the complementation vector in LB medium with and without the addition of 10 mM IPTG and isolated total bacterial RNA. Each of the RNA samples was then transcribed to obtain cDNA using reverse transcriptase and PCR amplification was performed using primers specific for the *bpaC* gene region downstream of the single cross-over insertion as detailed in the Materials and Methods. Densitometry analysis of the resulting PCR products indicated a low level of the *bpaC* transcript was present in the non-induced sample (0.75 of the value obtained for the 15 ng gDNA control); whereas, addition of IPTG resulted in an approximately 4-fold increase of the *bpaC* transcript (2.75 relative to the gDNA control). Thus, expression of the *bpaC* gene from the complementation vector occurs effectively under IPTG induction.

It has previously been reported that an independent *bpaC* mutant demonstrated attenuation for virulence in the BALB/c intra-nasal mouse model [15], suggesting that *bpaC* plays a role in *B*.*pseudomallei* pathogenesis. Our data presented here further support this notion and indicate that this AT may do so by contributing to serum resistance and intracellular survival of the pathogen.

### BpaE and BcaA are capable of inducing humoral and cell-mediated immune responses

**Fig 4:**
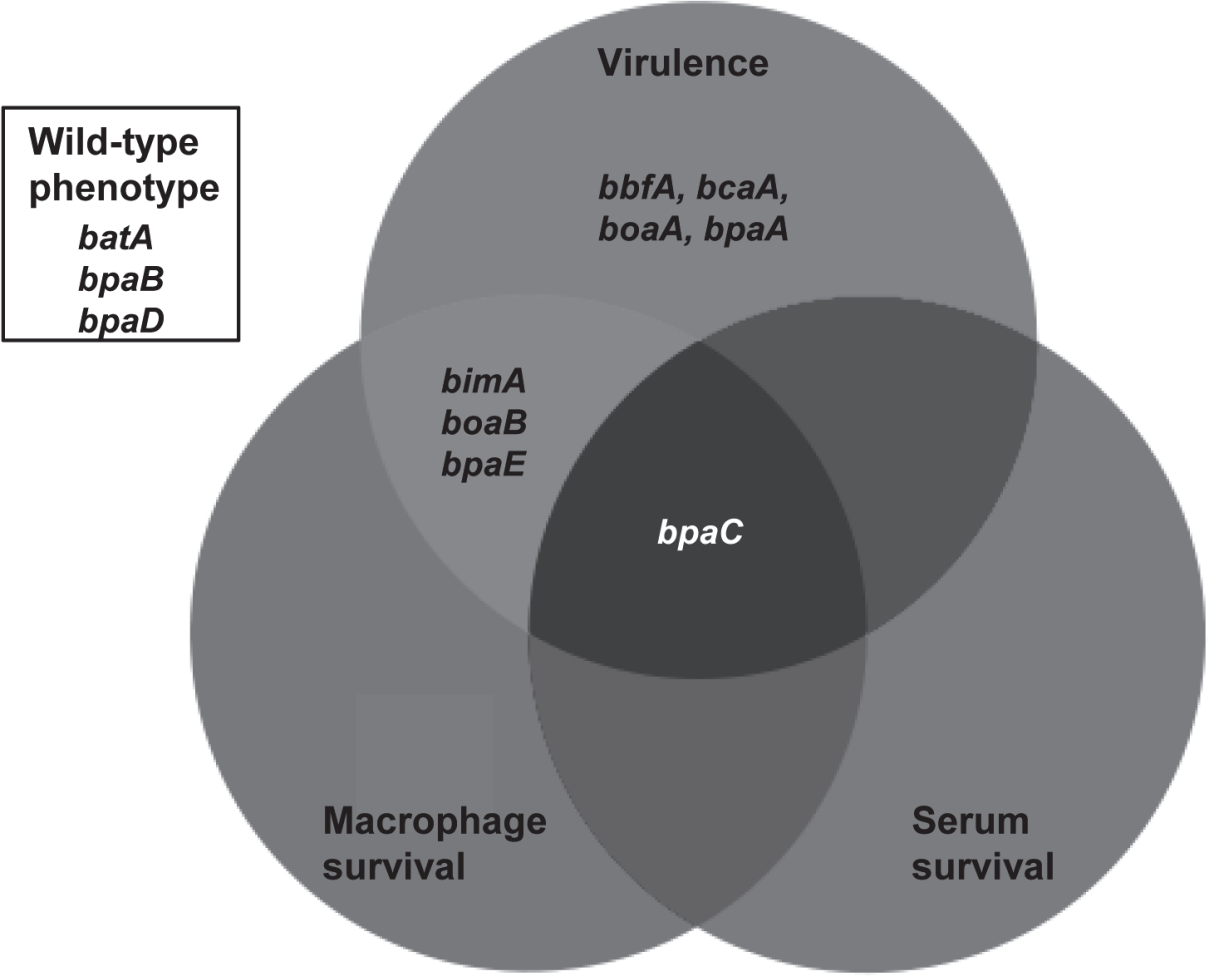
Systematic assessment of ATs for three pathogenesis-associated phenotypes. A Venn diagram of the phenotypic results of the complete repertoire of ATs for virulence in the BALB/c intra-peritoneal mouse melioidosis model, net intracellular replication in J774.2 macrophage-like cells and survival in 45% (v/v) normal human serum.

Seven TAAs have been shown to be recognised by melioidosis patient sera antibodies in two separate screens, indicative of *in vivo* expression and immunogenicity of these ATs ([16,20]; Table 3). Thus, we expressed recombinant N-terminal GST fusion proteins of the passenger domain of four predicted ATs to be used as antigens to test the immune response against these ATs. All recombinant proteins were examined by Western blot using anti-GST antibodies and confirmed to be of expected size; no degradation products were detected (data not shown). These four ATs represented each of the phenotypic groups identified in the three pathogenesis-associated assays detailed above (Fig. 4): those attenuated for virulence in the BALB/c intra-peritoneal mouse melioidosis model (BcaA), those attenuated for virulence and net intracellular replication in J774.2 macrophage-like cells (BpaE), the BpaC mutant with defects in virulence, net intracellular replication and serum resistance in 45% (v/v) normal human serum and those displaying wild-type phenotypes (BatA). This includes both classical ATs (BcaA and BatA) and two of the predicted TAAs (BpaE and BpaC). Only one of these antigens, BpaE, has been previously shown to be recognized by host immunity, although BpaC-specific antibodies have been detected in response to infection of BALB/c mice with *B*. *pseudomallei* [18].

**Fig 5:**
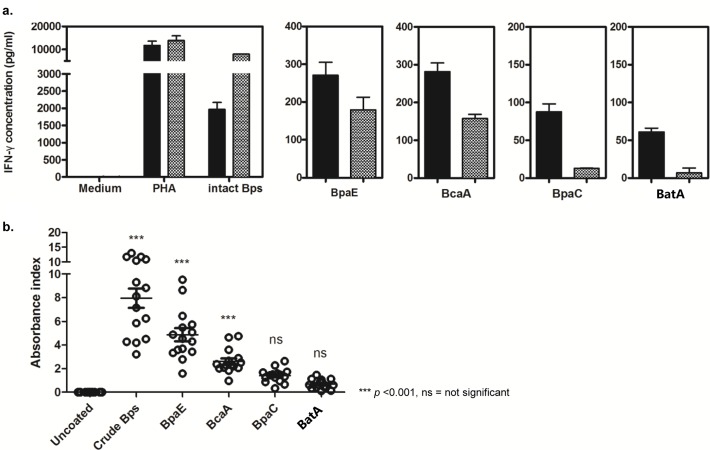
Immune recognition of recombinant proteins. a) Recombinant N-terminal GST fusion proteins of the passenger domain of four ATs were used as antigens (1 μg/ml) to stimulate whole blood (WB) from human seropositive donors, alongside PHA and fixed *B*. *pseudomallei* strain K96243 positive controls. The IFN-γ response of WB was measured by ELISA. BpaE and BcaA (225.4 pg/ml ± 45.7 pg/ml and 214 pg/ml ± 56.7 pg/ml respectively; p value <0.001) relative to the medium-only control average (4.99 pg/ml ± 1.7 pg/ml) elicited a strong IFN-γ response from WB from seropositive donors (n = 2; black and grey bars represent the two individual donors) while BpaC and BatA did not induce a significant lymphocyte recall response. b) The four antigens were also tested for recognition by human seropositive sera from a melioidosis endemic area using an indirect sandwich ELISA. BpaE and BcaA (absorbance index of 7.96 ± 0.82 and 4.86 ± 0.55 respectively; *p* value <0.001) were both recognised by seropositive human sera from the endemic area while values for neither BpaC nor BatA were significantly different from the negative control.

Soluble recombinant N-terminal GST fusion proteins were used as antigens to test the immune response against ATs. The four antigens (1 μg/ml) were used to stimulate whole blood from human seropositive donors, alongside mitogen (PHA) and fixed *B*. *pseudomallei* K96243 positive controls as detailed in Materials and Methods. The IFN-γ response of whole blood from seropositive donors was measured by ELISA and is indicative of lymphocyte reactivation to the antigens tested. BpaE and BcaA elicited a strong average IFN-γ response across the donors (225.4 pg/ml ± 45.7 pg/ml and 214 pg/ml ± 56.7 pg/ml respectively; *p* value <0.001) relative to the medium-only control average (4.99 pg/ml ± 1.7 pg/ml) while BpaC and BatA did not induce a statistically significant lymphocyte recall response across the donors (Fig. 5A).

The four antigens were also tested for recognition by seropositive sera from an area where melioidosis is endemic using an indirect sandwich ELISA as detailed in Materials and Methods. BpaE and BcaA (absorbance index of 7.96 ± 0.82 and 4.86 ± 0.55 respectively; *p* value <0.001) were both recognised by seropositive human sera from the endemic area while values for neither BpaC nor BatA were significantly different from the negative control (Fig. 5B).

The immunogenicity data demonstrates that two of the ATs tested (BpaE and BcaA) are capable of eliciting humoral and cell-mediated responses in human melioidosis patients. Only one of these two ATs, BpaE, has been previously identified as immunogenic [16,20]; however, our study used a different experimental method to both previous studies so it is unsurprising that different antigens were identified. Neither BpaC nor BatA elicited a consistent lymphocyte recall response nor were these antigens recognised by seropositive patient serum, at least at the limit of detection of the assays used. BpaC has been previously demonstrated to be expressed *in vivo* by *B*. *pseudomallei* in BALB/c mice during acute infection [18], but if BpaC-specific antibodies were produced by the human melioidosis patients, they were not detected by our assays. This may relate to a degree of variation noted in domain repeats of BpaC across *B*. *pseudomallei* strains [18], or reflect differences in the immune response between hosts (murine versus human). In the case of the BatA, when these data are taken together with the fact that the *bpaB* mutant displays wild-type virulence and net intracellular replication, it suggests that this AT may not be highly expressed *in vivo*.

To conclude, we have systematically mutated eleven predicted ATs of *B*. *pseudomallei* strain 10276 and assigned roles to eight of these in the pathogenesis of melioidosis in a murine intra-peritoneal challenge model, of which four also contributed significantly to net intracellular replication in macrophage-like cells *in vitro*. We also identified a novel serum resistance factor, BpaC, which mediates resistance to killing by the classical and/or lectin arms of the complement pathway. Further studies are required in order to elucidate the mechanism of BpaC-mediated serum resistance and to determine the value of virulence-associated and immunogenic ATs in the development of strategies to control melioidosis.

## Supporting Information

S1 Table Oligonucleotide used. (DOCX)

S1 Fig The *bpaC* mutant expresses polysaccharide capsule and normal LPS **a) The *bpaC* mutant and its parent wild-type 10276 strain were assessed for the presence of capsular material by immunofluorescence microscopy.** Mid-log cultures of the *B*. *pseudomallei* strains were stained with the type I O-PS capsule monoclonal antibody 4V1H12. Stained bacteria were observed using immunofluorescence microscopy and both strains demonstrated capsular material.b) The *bpaC* mutant and its parent wild-type 10276 strain were assessed for the presence of LPS by silver staining and immunoblotting. The LPS of overnight cultures of the *B*. *pseudomallei* stains was visualised by silver staining and immunoblotting with anti-*B*. *mallei* LPS monoclonal antibody. Both strains demonstrated similar LPS profiles with clearly visible O-Ag.
(TIF)
