# Supplementary material for: Systematic Mutagenesis of Genes Encoding Predicted Autotransported Proteins of Burkholderia pseudomallei Identifies Factors Mediating Virulence in Mice, Net Intracellular Replication and a Novel Protein Conferring Serum Resistance
Source: PLoS One. 2015 Apr 1;10(4):e0121271. doi: 10.1371/journal.pone.0121271 (PMC4382181; doi:10.1371/journal.pone.0121271)
Supplement: S1 Table — (DOCX) [file pone.0121271.s001.docx]

**S1 Table: Oligonucleotide used.**

| **Gene** | **Forward and reverse primer pairs (5’… 3’ sequence) ⱡ** |
| --- | --- |
| *Single cross-over mutagenesis constructs* | |
| *bpaC* | TGCTCGAGCTGTCGATTCTGGGCGCGGCAT |
|  | CGAGAGATCTTGCGTCACGACGCCCAC |
| *boaA* | CGGCTCGAGCGGCGGCGGCGGTGTCCAGTT |
|  | GCCGTAGATCTCAGGCCGGTCGCGTGAGCGCCG |
| *bpaE* | CACGCTCGAGTACGGCACCGCGGTCGG |
|  | CCGAGATCTTTCGCGTAATAGCCGGACGAT |
| *bcaA* | TCCCTCGAGTTGGCGCACGCCGGAATTCAC |
|  | GCGAAGATCTTCGCATCCGACGCGGGAA |
| *bpaD* | AGCAACTCTAGAGCAACGGGGCAGGATGCTAA |
|  | GCTTTGGAATTCCTGCGCCGAGGCATTGCC |
| *batA* | GCGGCCGGATCCTACACGTTCTATGCGCTCGG |
|  | GCTCGCTCTAGACAGGTCCTGATCGTTGGTG |
| *bpaB* | CTGCTTGGATCCCCTGCGTGGGCGGACA |
|  | CGTCACTCTAGATGTTGGTCCCCGCATACT |
| *bpaA* | ATCTCGGGATCCCGCGGCAAGCCGAACAAGTC |
|  | GCTAGTTCTAGAGCTCATGCCCGTACTCAGTT |
| *boaB* | CCGGCTGGATCCGGTACGGTAATAGGCGGCGG |
|  | AAGCTGTCTAGATTATTGAGCCCGAGCGTACT |
| *Recombinant N-terminal GST-fusion passenger domain protein expression constructs* | |
| *bpaC* | GACGTAGGATCCGGTGGCCGCGCGTCGA |
|  | CCCGCTGAATTCGCTGTTGTTGCCCGACGCAG |
| *bpaE* | GACGCCGGATCCCAGGTCAGCTATGCGGC |
|  | AATCGCCCCGGGCAACTGACCGACGTTTACCG |
| *bcaA* | CTTGTAAGATCTCAGGCCGCGCCGTACCCGGAT |
|  | CCGGATGATATCTAGAATAGCCGCTCGGCGCATCGA |
| *batA* | GCGGCCGGATCCTACACGTTCTATGCGCTCGG |
|  | TAGCATGATATCCCACGCGACGCGCGCTGG |
| *RT-PCR specific primers* | |
| *bpaC fwd* | GTCGGAGGACAACACGGTAT |
| *bpaC rve* | CCGTCTTCACCTTGAGGTTC |

ⱡ Engineered restriction sites are underlined.
