# Supplementary figures and images for: Systematic Mutagenesis of Genes Encoding Predicted Autotransported Proteins of Burkholderia pseudomallei Identifies Factors Mediating Virulence in Mice, Net Intracellular Replication and a Novel Protein Conferring Serum Resistance
Source: PLoS One. 2015 Apr 1;10(4):e0121271. doi: 10.1371/journal.pone.0121271 (PMC4382181; doi:10.1371/journal.pone.0121271)

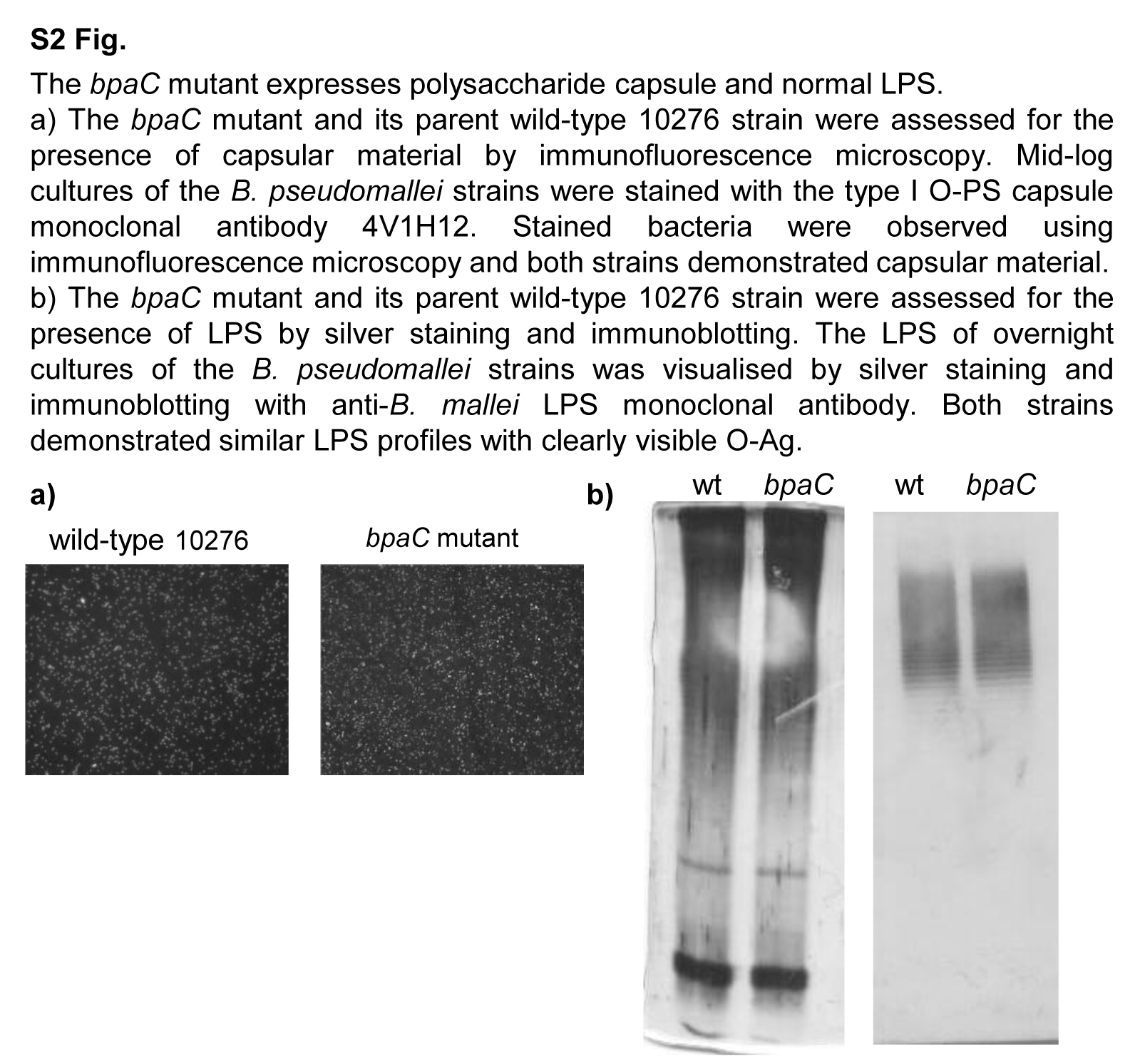

Supplement: S1 Fig — a) The bpaC mutant and its parent wild-type 10276 strain were assessed for the presence of capsular material by immunofluorescence microscopy. Mid-log cultures of the B. pseudomallei strains were stained with the type I O-PS capsule monoclonal antibody 4V1H12. Stained bacteria were observed using immunofluorescence microscopy and both strains demonstrated capsular material.b) The bpaC mutant and its parent wild-type 10276 strain were assessed for the presence of LPS by silver staining and immunoblotting. The LPS of overnight cultures of the B. pseudomallei stains was visualised by silver staining and immunoblotting with anti-B. mallei LPS monoclonal antibody. Both strains demonstrated similar LPS profiles with clearly visible O-Ag. (TIF) [file pone.0121271.s002.tif]
